# The Microstructural Evolution and Mechanical Properties of Zr-Based Metallic Glass under Different Strain Rate Compressions

**DOI:** 10.3390/ma8041831

**Published:** 2015-04-16

**Authors:** Tao-Hsing Chen, Chih-Kai Tsai

**Affiliations:** Department of Mechanical Engineering, National Kaohsiung University of Applied Sciences, Kaohisung 807, Taiwan

**Keywords:** Zr-based bulk metallic glass, buckling, shear band, *in situ* TEM

## Abstract

In this study, the high strain rate deformation behavior and the microstructure evolution of Zr-Cu-Al-Ni metallic glasses under various strain rates were investigated. The influence of strain and strain rate on the mechanical properties and fracture behavior, as well as microstructural properties was also investigated. Before mechanical testing, the structure and thermal stability of the Zr-Cu-Al-Ni metallic glasses were studied with X-ray diffraction (XRD) and differential scanning calorimeter. The mechanical property experiments and microstructural observations of Zr-Cu-Al-Ni metallic glasses under different strain rates ranging from 10^−3^ to 5.1 × 10^3^ s^−1^ and at temperatures of 25 °C were investigated using compressive split-Hopkinson bar (SHPB) and an MTS tester. An *in situ* transmission electron microscope (TEM) nanoindenter was used to carry out compression tests and investigate the deformation behavior arising at nanopillars of the Zr-based metallic glass. The formation and interaction of shear band during the plastic deformation were investigated. Moreover, it was clearly apparent that the mechanical strength and ductility could be enhanced by impeding the penetration of shear bands with reinforced particles.

## 1. Introduction

Bulk metallic glass (BMG) and its composites (BMGC) are of interest due to their unique mechanical, physical, and chemical properties, such as amorphous microstructure, ultra-high strength, large limit of elastic deformation, and excellent corrosion resistance [1,2,3,4,5]. The properties of amorphous alloys are quite different from those of crystalline metal alloys. BMG suffers from highly localized shear deformation at room temperature under sufficiently high applied pressure [6,7]. During indentation, compression, or tension tests, the formation of shear bands leads to brittle fractures along the shear planes, and the resulting lack of ductility greatly limits the application of BMG. Reinforcements such as refractory metals, fibers, and ceramic particles have thus been added into BMG matrices to enhance their ductility [8,9,10,11]. Some researchers report that the size and geometry of the specimens have significant influences on the ductility and fracture behavior of BMGs [12,13]. The measured mechanical properties of BMG vary with specimen shape, due to the effects of both size, geometry and stress concentration at the sample/platen interface [14,15,16].

Since Zr-based metallic glass is used in structural materials, it is important to examine the related mechanical properties under different strain rate conditions, although there are few studies that have discussed this issue. Many studies have investigated the basic amorphous forming ability of metallic glass and the tension fracture mechanical behavior [17,18,19], but very few have investigated the effects of the strain rate on the mechanical properties and fracture characteristics. This work utilized an MTS tester and split-Hopkinson bar to investigate the effects of strain rate on Zr-based metallic glass.

*In situ* transmission electron microscope (TEM) compression/tension experiments were recently used to determine the mechanical responses of BMGs at the nano-scale [20,21]. A numbers of studies investigated the influence of size [22,23,24,25,26,27,28,29,30,31,32] (*i.e.*, nanopillars diameter) on the deformation mode. Furthermore, this study also used an *in situ* transmission electron microscope (TEM) nanoindenter to carry out compression tests and investigate the related deformation behavior (ex. shear banding and buckling deformations) arising at nanopillars of the Zr-based metallic glass.

## 2. Material Preparation and Experimental Procedure

A Zr-based metallic glass ingot (Zr_53_Cu_30_Ni_9_Al_8_) was selected as the base alloy for the preparation of BMG specimens. High purity Zr, Cu, Ni, and Al were melted together using an arc melting system in an argon (Ar) atmosphere. The alloy ingot was fabricated by injecting the material into a water-cooled copper mold to obtain amorphous alloy rods. The cylindrical rods were fabricated with a diameter of 4 mm and length of 4 mm. X-ray diffraction and electron backscatter diffraction (EBSD) were then used to measure the crystalline structure of the rods. Hydrostatic tests were carried out using a compressive MTS tester at strain rates of 10^−1^ s^−1^, 10^−2^ s^−1^ and 10^−3^ s^−1^, and dynamic impact tests were carried out using a compressive split-Hopkinson bar (SHPB) at strain rates of 2.0 × 10^3^ s^−1^, 3.0 × 10^3^ s^−1^ and 5 × 10^3^ s^−1^. The fracture surface observations were examined using scanning electron miocroscopy (SEM).

**Figure 1:**
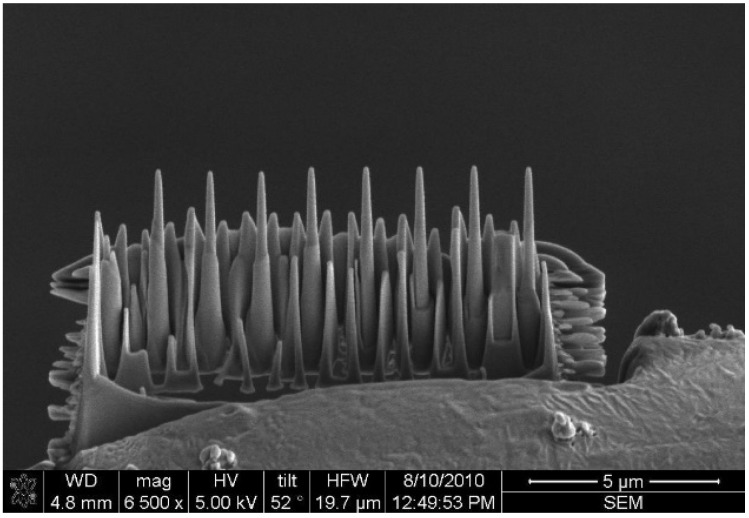
Zr-based metallic glass nanopillars fabricated by dual-beam focused ion beam (DB-FIB) for *in situ* transmission electron microscopy (TEM) compression experiments.

*In situ* TEM compression experiments were performed using a Hysitron Picoindenter TEM holder (Hysitron, Minneapolis, MN, USA) embedded in a JEOL 2010 TEM. The indenter used in the compression experiment was a diamond flat punch, two μm in diameter. The compression tests of the nanopillars were performed using displacement control. In displacement control mode, the maximum punch displacement was 500 nm and the loading/unloading time was 10 seconds. A dual-beam focused ion beam (FEI, Hillsboro, OR, USA) with an *in situ* pickup system (Omni-Probe) was then used to fabricate the Zr-based metallic glass nanopillars, as shown in Figure 1.

## 3. Results and Discussion

### 3.1. Structure Analysis of Zr-Based Metallic Glass

**Figure 2:**
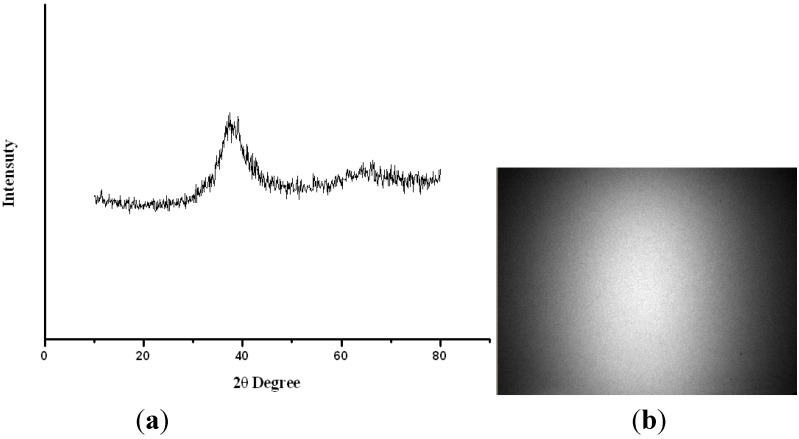
(**a**) X-ray diffraction (XRD) pattern; (**b**) The electron backscatter diffraction (EBSD pattern of the as-cast Zr-based metallic glass.

Figure 2a shows the X-ray diffraction (XRD) patterns of the Zr-based metallic glass, and it also show that the as-cast specimens are amorphous, with a broadened and diffused hump under diffraction in the 2θ range. No crystalline structures can be seen. The EBSD results, as shown in Figure 2b, also show the absence of any crystalline phase.

### 3.2. Stress-Strain Curve and Strain Rate Effect

**Figure 3:**
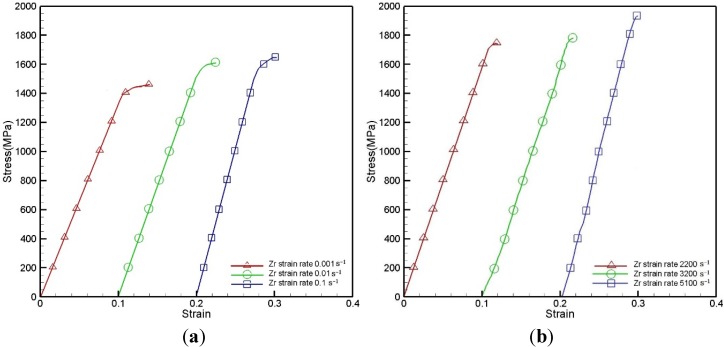
True stress-strain curves of Zr-based metallic glass deformed at strain rates of (**a**) 10^−3^, 10^−2^ and 10^−1^ s^−1^; (**b**) 2.2 × 10^3^, 3.2 × 10^3^ and 5.1 × 10^3^ s^−1^.

Figure 3a presents the typical stress-strain curve of Zr-based metallic glass at 25 °C and under quasi-static strain rates of 10^−3^ s^−1^, 10^−2^ s^−1^ and 10^−1^ s^−1^. From Figure 3a, it can be seen that the yield stress increases along with the strain rate, while the fracture strain decreases. Furthermore, the total fracture strain of each strain rate condition is very small. Serrations can be clearly observed after yielding, due to the plastic flow of shear bands during the loading process. Figure 3b shows the stress-strain behavior with high strain rates of 2.2 × 10^3^ s^−1^, 3.2 × 10^3^ s^−1^ and 5.1 × 10^3^ s^−1^. It can also be observed that the yield stress increases along with the strain rate. Comparing the quasi-static and dynamic condition, the yield strength increases from 10^−3^ to 5.1 × 10^3^ s^−1^. Table 1 shows the yield stress under different strain rates. The serrations are not formed at the high strain rate condition, and the fracture strain also becomes smaller.

**Table 1:** Yield stress of Zr-based metallic glass under different strain rates.

Strain rate (s^−1^)	Yielding stress (MPa)
10^−3^	1400
10^−2^	1580
10^−1^	1600
2.2 × 10^3^	1720
3.2 × 10^3^	1785
5.1 × 10^3^	1840

The stress-strain relations observed in Figure 3 clearly show that the stress of Zr-based metallic glass is significantly dependent on the strain rate. The strain rate sensitivity (β) of the Zr-based metallic glass can be derived from the experimental results presented in Figure 3 in accordance with the following equation [33].

(1) $$\beta =(\partial \sigma /\partial \ln\dot{\varepsilon })=\frac{\sigma_{2}-\sigma_{1}}{\ln({\dot{\varepsilon }}_{2}/{\dot{\varepsilon }}_{1})}$$

where the compressive stresses *σ*_2_ and *σ*_1_ relate to impact tests conducted at average strain rates of ${\dot{\varepsilon }}_{2}$ and ${\dot{\varepsilon }}_{1}$, respectively, and are calculated at the same value of plastic strain.

**Figure 4:**
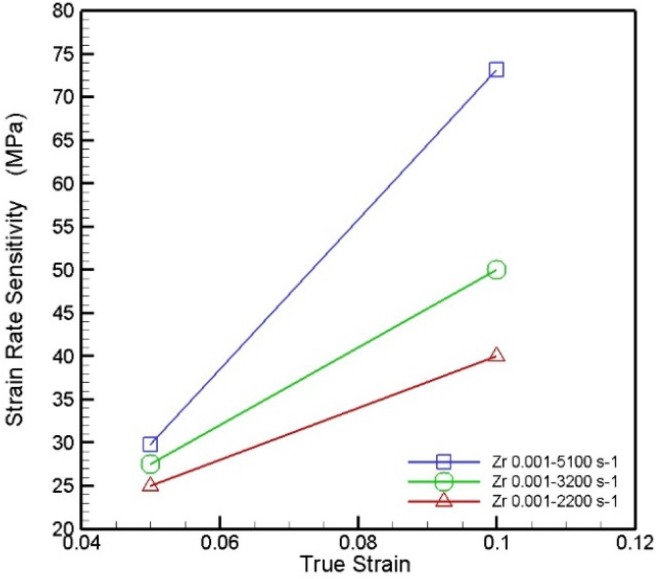
Variation of strain rate sensitivity with true strain as function of strain rate.

Figure 4 plots the strain rate sensitivity of the Zr-based metallic glass as a function of the true strain for two different strain rate ranges at a temperature of 25 °C. It can be seen that the strain rate sensitivity increases with increasing strain and strain rate, and that the sensitivity increases particularly rapidly at higher strain rates (*i.e.*, 10^−3^ to 5.1 × 10^3^).

### 3.3. Fracture Surface Observations

**Figure 5:**
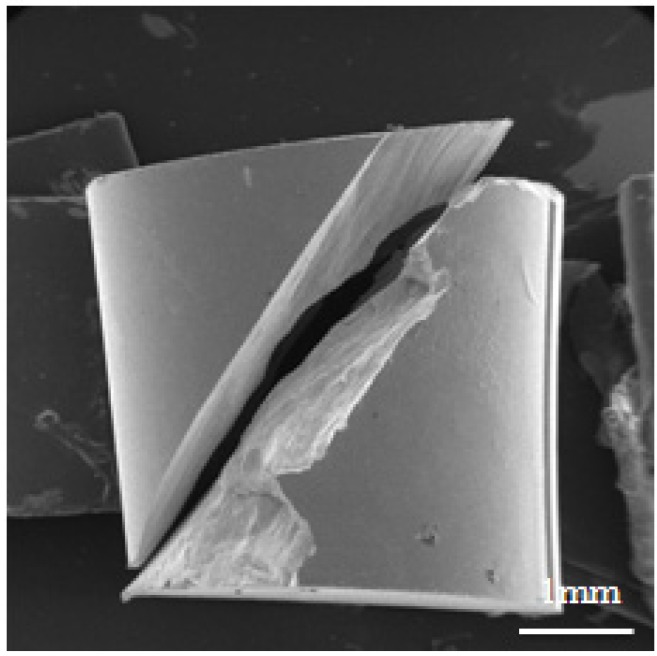
The SEM fractograph of Zr-based metallic glass deformed at a strain rate of 2.2 × 10^3^ s^−1^.

**Figure 6:**
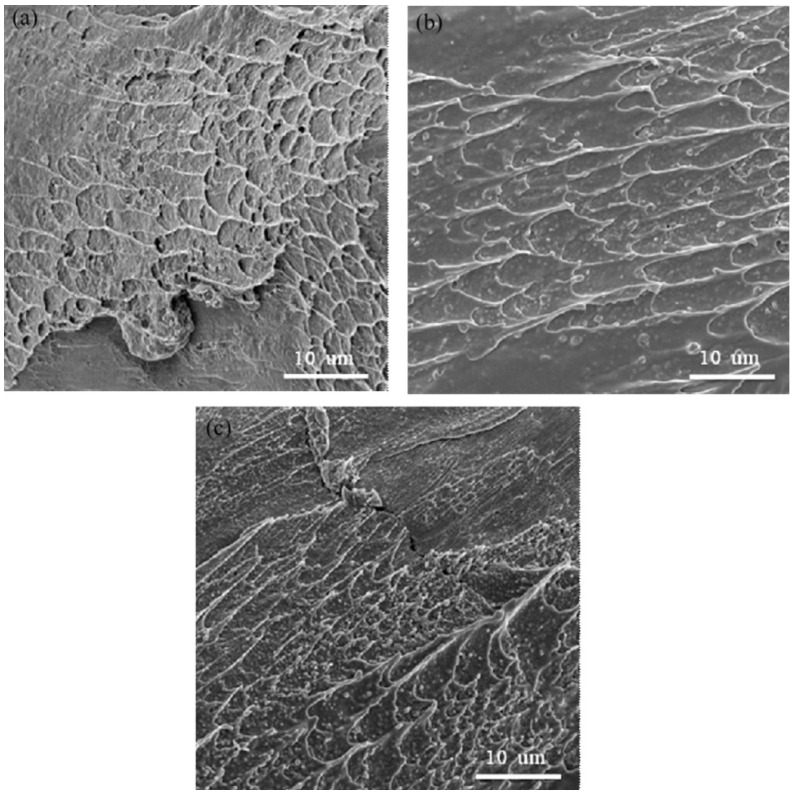
SEM fracture surface of Zr-based metallic glass specimens deformed at strain rates of (**a**) 10^−3^ s^−1^; (**b**) 10^−2^ s^−1^; and (**c**) 10^−1^ s^−1^.

**Figure 7:**
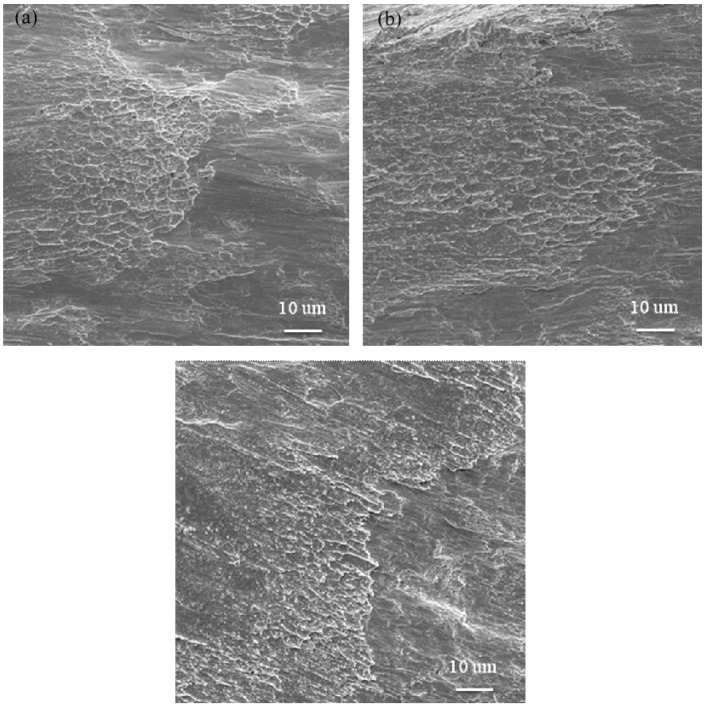
SEM fracture surface of Zr-based metallic glass specimens deformed at strain rates of (**a**) 2.2 × 10^3^ s^−1^; (**b**) 3.2 × 10^3^ s^−1^; and (**c**) 5.1 × 10^3^ s^−1^.

Figure 5 shows a side view SEM fractograph of a specimen tested at a strain rate of 2.2 × 10^3^ s^−1^ and temperature of 25 °C. It can be seen that the specimen failed along a plane orientated at 45° to the loading direction, and a few shear bands can be observed on the fracture surface. Figure 6a–c presents the fracture surface of Zr-based metallic glass at strain rates of 10^−3^ s^−1^ to 10^−1^ s^−1^. It can be observed that the fracture surface has a vein-like structure [34], and this morphology indicates good plasticity [35,36]. Furthermore, the density of this vein-like structure decreases as the strain rate increases. The phenomenon corresponds with the results shown in Figure 3a, in which fracture strain decreases as the strain rate increases. Similar tendencies are noted in Figure 7a–c at strain rates of 2.2 × 10^3^ s^−1^ to 5.1 × 10^3^ s^−1^, respectively. It is also observed that the density of the vein-like structure on the fracture surface decreases as the strain rate increases. For example, comparing Figure 6 and Figure 7 it can be seen that the density of the vein-like structure decreases when the strain rate increases from 10^−3^ s^−1^ to 5.1 × 10^3^ s^−1^.

### 3.4. Microstructural Evolution by Using in situ TEM Compression

**Figure 8:**
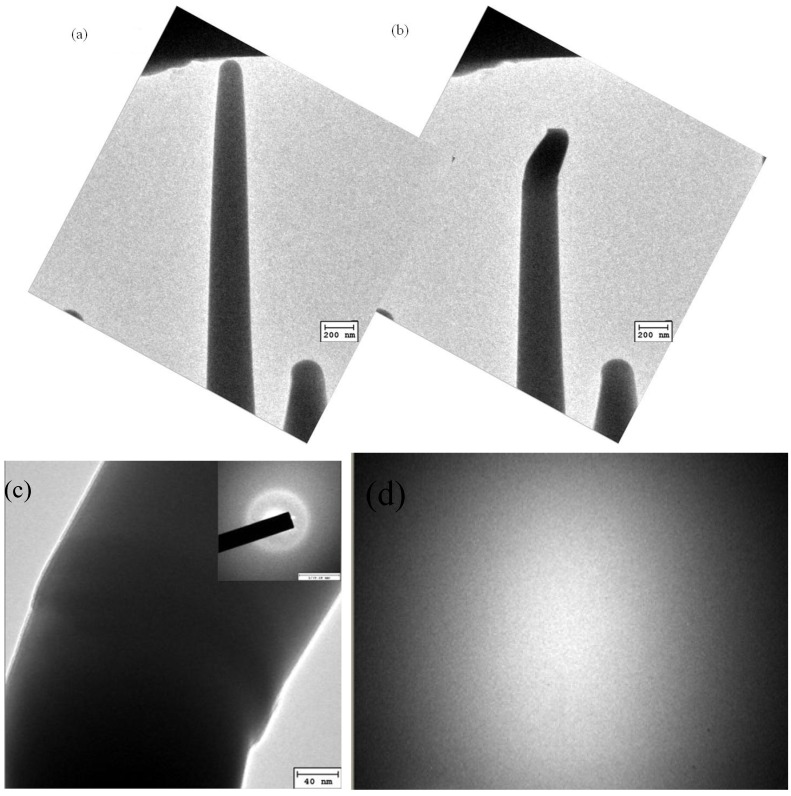
TEM bright field images of a Zr-based metallic glass nano-pillar (**a**) before, and (**b**) after the *in situ* TEM nano-compression experiment; (**c**) the buckling position of a Zr-based metallic glass nano-pillar; (**d**) the EBSD pattern of Zr-based metallic glass after high strain rate deformation.

Figure 8a shows the nano-pillar and the indenter before the application of compression. During compression, buckling occurs at a compression depth of 120 nm, as shown in Figure 8b. Figure 8c presents a higher magnification of the TEM bright field images in the buckling position for the specimens. Buckling behavior is presented in the form of semi-homogeneous deformation. After compression, the microstructures of the buckling region are still in the amorphous phase. It is thus known that the deformation behavior of the Zr-based metallic glass did not include any phase transformation, and deformation only occurred in the propagation of shear bands. Figure 8d shows the EBSD figure of the specimen with a strain rate of 5.1 × 10^3^ s^−1^. It also can be seen that even though the Zr-based metallic glass deformed at a high strain rate, it still kept its amorphous phase.

## 4. Conclusions

This study conducted an experimental investigation into the effects of strain rate on the mechanical properties and microstructural evolution of Zr-based metallic glass at room temperature and at strain rates ranging from 10^−3^ s^−1^ to 5.1 × 10^3^ s^−1^ using an MTS tester and split-Hopkinson bar. The experimental results show that the yield stresses all for all the tested specimens increase along with the strain rate. Moreover, when the strain rate increases the strain rate sensitivity also rises. The SEM observations show that the fracture surfaces are characterized by a vein-like structure, which indicates that the Zr-based metallic glass specimens have good plasticity. Furthermore, the results of the *in situ* TEM experiment show that the Zr-based metallic glass maintains an amorphous phase during the deformation process.
